# Electronic Health Use in a Representative Sample of 18,497 Respondents in Norway (The Seventh Tromsø Study - Part 1): Population-Based Questionnaire Study

**DOI:** 10.2196/13106

**Published:** 2020-03-05

**Authors:** Rolf Wynn, Sunday Oluwafemi Oyeyemi, Andrius Budrionis, Luis Marco-Ruiz, Kassaye Yitbarek Yigzaw, Johan Gustav Bellika

**Affiliations:** 1 Department of Clinical Medicine Faculty of Health Sciences UiT The Arctic University of Norway Tromsø Norway; 2 Division of Mental Health and Addictions University Hospital of North Norway Tromsø Norway; 3 Department of Community Medicine Faculty of Health Sciences UiT The Arctic University of Norway Tromsø Norway; 4 Norwegian Centre for E-health Research University Hospital of North Norway Tromsø Norway

**Keywords:** adoption, digital health, eHealth, internet, Web search engine, health apps, social media, video service, population study, Tromsø study

## Abstract

**Background:**

Electronic health (eHealth) services may help people obtain information and manage their health, and they are gaining attention as technology improves, and as traditional health services are placed under increasing strain. We present findings from the first representative, large-scale, population-based study of eHealth use in Norway.

**Objective:**

The objectives of this study were to examine the use of eHealth in a population above 40 years of age, the predictors of eHealth use, and the predictors of taking action following the use of these eHealth services.

**Methods:**

Data were collected through a questionnaire given to participants in the seventh survey of the Tromsø Study (Tromsø 7). The study involved a representative sample of the Norwegian population aged above 40 years old. A subset of the more extensive questionnaire was explicitly related to eHealth use. Data were analyzed using logistic regression analyses.

**Results:**

Approximately half (52.7%; 9752/18,497) of the respondents had used some form of eHealth services during the last year. About 58% (5624/9698) of the participants who had responded to a question about taking some type of action based on information gained from using eHealth services had done so. The variables of being a woman (OR 1.58; 95% CI 1.47-1.68), of younger age (40-49 year age group: OR 4.28, 95% CI 3.63-5.04), with a higher education (tertiary/long: OR 3.77, 95% CI 3.40-4.19), and a higher income (>1 million kr [US $100,000]: OR 2.19, 95% CI 1.77-2.70) all positively predicted the use of eHealth services. Not living with a spouse (OR 1.14, 95% CI 1.04-1.25), having seen a general practitioner (GP) in the last year (OR 1.66, 95% CI 1.53-1.80), and having had some disease (such as heart disease, cancer, asthma, etc; OR 1.29, 95% CI 1.18-1.41) also positively predicted eHealth use. Self-rated health status did not significantly influence eHealth use. Taking some action following eHealth use was predicted with the variables of being a woman (OR 1.16, 95% CI 1.07-1.27), being younger (40-49 year age group: OR 1.72, 95% CI 1.34-2.22), having a higher education (tertiary/long: OR 1.65, 95% CI 1.42-1.92), having seen a GP in the last year (OR 1.58, 95% CI 1.41-1.77), and having ever had a disease (such as heart disease, cancer or asthma; OR 1.26, 95% CI 1.14-1.39).

**Conclusions:**

eHealth appears to be an essential supplement to traditional health services for those aged above 40 years old, and especially so for the more resourceful. Being a woman, being younger, having higher education, having had a disease, and having seen a GP in the last year all positively predicted using the internet to get health information and taking some action based on this information.

## Introduction

Online resources, including the use of search engines, social media, apps, and online video services, are becoming increasingly important for people in their everyday lives [1,2]. For example, 84% of US adults use the internet [3]. In the European Union (EU) in 2012, 73% of the population were online [4], and in Norway, in 2013, this was the case with 85% of the population [5]. When it comes to the specific use of social media, in 2016, a total of 68% of US adults used Facebook [6].

Electronic health (eHealth) is the use of Information and Communication Technology, such as the internet, to enable or improve health care [7]. While other eHealth activities, such as using health apps to make appointments and order prescriptions and using social media for communicating with health professionals, are becoming more popular, by far, the most frequent eHealth activity is finding information about health and illness on the internet [8-12]. Approximately 77% of online health searches start at a search engine [13]. In 2012, 59% of Americans had searched the internet for health information [12], and in 2013, 78% of Norwegians had used the internet for health purposes [8]. Prior studies have suggested that being young, being a woman, and being highly educated are central predictors of eHealth use [13]. A prior study found that 19% of smartphone users had at least one health app, and this was more frequent among women, those of a younger age, those with high incomes, and the college-educated [14]. One study found that 35.7% of all seniors and 89.1% of all those that were online used Facebook, Twitter, etc, to find and share health information [10]. Being a woman and highly educated predicted social media use for health information [10]. People with a primary health care provider, chronic disease, and of a younger age have been found to be more likely to use social media for health [11]. Thus, prior research has established that searching for health information is, overall, the most frequent eHealth activity. We also know that some demographic groups have been found to be more active online health-information searchers, but we lack updated information on this topic from a Nordic setting.

The aging population in many Western countries is likely to increase demands on health services. An increasing number of people with chronic illnesses are likely to stretch the capacity of health services further, and as many as 45% of US adults have one or more chronic illnesses [15]. eHealth may add to traditional services by engaging patients, helping patients to get information, empowering patients, increasing shared decision-making, and helping patients manage their health [16-21]. eHealth literacy is the competency required to use and make sense of eHealth tools and services [22], and it has been associated with younger age, a higher education, and having more devices [10]. eHealth has also been suggested as a means whereby shortages in health care staff and funding can be addressed (ie, the current crisis in the British National Health Service) [23]. Furthermore, eHealth, in combination with good eHealth literacy, might reduce the currently elevated number of unnecessary visits to doctors [24]. Thus, prior research has suggested that eHealth could help empower patients [25], and it has also been suggested that eHealth could help address resource shortages in the traditional health services, but the effect of eHealth use on traditional health service consumption has not been well established in the Nordic countries.

It remains unclear how eHealth influences traditional health service use in Norway, whether eHealth tools and services can replace traditional services or whether eHealth tools and services should be added to existing health service use [26]. Studies from other countries have found different results. One study found that 35% of US adults had gone online to figure out a medical condition and that 53% of these followed up with a visit to a medical professional [12]. Online self-diagnosing was more common among younger white adults, high earners, and the highly educated compared to others [12]. Other studies in the United States, Japan, and Taiwan have found that the use of the internet either increased or had no significant influence on traditional health care use [27-32]. For instance, Lee [27] found that increased internet use at survey wave one positively predicted health professional contact at survey wave two. Ybara and Suman [28] found that an increase in internet use increased the chance of the respondent visiting a physician. Hisieh et al [29], in a Taiwanese study controlling for a range of variables, including sex and age, found that increased internet use and chronic illness both predicted increased outpatient visits. Baker et al [31] found that female gender, younger age, higher education, and worse health were all variables that predicted increased internet use for health information searching but found that internet use had little influence on the use of traditional health professional consultations. Takahashi et al [32] found that younger age, higher education, and higher income were associated with an increase in searches for online health information but found no significant influence of online research on general practitioner (GP) visits. Interestingly, one Dutch study found a reduction in the use of traditional health services after the introduction of a high-quality, online health-information service [33]. More research is needed to explore how the use of eHealth will impact the use of traditional health services (such as GP visits) in Norway.

While prior studies have suggested that there are social divisions in the use of the internet for health purposes, many of these have been based on web-panels and other samples that might not have been fully representative of the general population [34]. In Europe, one major study included patients from seven countries [35], with a total of 8000 respondents. The study showed that Northern European countries, such as Denmark and Norway, had higher rates of eHealth use than Southern European countries, such as Portugal and Greece. Among those online, young adults, women, and the highly educated, as well as those who had visited a GP in the last year, and those who had a chronic illness more often searched online for health information. The study was carried out in 2005 and used random dialing within strata to sample respondents. A survey of 13,000 Europeans participating in an online panel found that younger adults, females, those living in larger households, those who had children or elderly family members, those with health problems, and those that were caring for others had a higher propensity towards intensive eHealth use [36]. Smaller studies from Europe of health information searching on the internet have suggested rates of searching are increasing across the continent [1,37,38].

In the United States, at least two larger studies have used representative samples: the Health Information National Trends Survey (HINTS) study [9,13,39,40] and the Pew Internet Study [3,6,12,14,15]. However, their findings might not be directly applicable to Western Europe, where services are organized differently and funded differently than in the United States. While the government, using taxation, funds nearly all health care in Nordic countries and many European countries (such as the United Kingdom), health care in the United States is typically based on different types of insurance. This means that while all citizens, regardless of their financial situation, have (at least in principle) equal access to health care in the Nordic model, access to and use of health services in the United States depends on an individual’s insurance coverage. While little is known about the importance of traditional health service organization on eHealth use, one could speculate that eHealth could see less use in a model where traditional services are more or less free (ie, the Nordic model). A representative, population-based study in a Nordic country will give more reliable data about the use of eHealth services in the general population in a setting with a free-for-all (ie, tax-funded), government-operated health service, including data on the possibly increasing importance of more recent sources of health information, such as social media, apps, and online video services [41-44].

Nordic countries, especially the subarctic regions, are sparsely populated, and access to specialist health services may be limited in rural areas. eHealth services could be particularly relevant for stakeholders and policymakers in sparsely populated, rural, and remote areas [45,46]. It is essential for health providers and decision-makers in the health services to know how new media impacts information seeking about health and illness. Knowledge about the use of eHealth services, including health information consumption, may be used to modify and target health information to specific groups and to establish whether existing services are tailored to current needs [9]. Age, gender, educational level, health status, and others are variables that are likely to influence the use of eHealth services, as these factors may be of importance to questions that arise around the ability to use these services. In this light, it becomes central to establish which resources are available and are used, who utilizes eHealth services and who does not, and to what extent eHealth services are replacing traditional health services.

The seventh, population-based Tromsø Study included a questionnaire about the use of eHealth. In a series of four papers, we explore data from this questionnaire and how the use of eHealth related to a range of other variables that were measured in the Tromsø Study. In the first paper (this paper), we present our main findings regarding the characteristics of the participants and their use of eHealth. In the present study, we examined the use of eHealth in the population above 40 years of age, predictors of eHealth use, and predictors of participants acting following their use of eHealth services. In the second paper [47] we will present and discuss how having different illnesses influences the use of eHealth, in the third paper [48] we will examine outcomes of the use of eHealth, and in the fourth paper [49] we will study how eHealth consumption influences actual doctor visits.

## Methods

### The Seventh Survey of the Tromsø Study

The Tromsø Study is a population-based, longitudinal health study conducted by the University of Tromsø in cooperation with several other Norwegian public agencies [50]. Inhabitants of the municipality of Tromsø were invited to participate in the study. Tromsø is the major city of North Norway, with a population of about 75,000 inhabitants. The seventh survey of the Tromsø Study (Tromsø 7) was conducted in 2015-2016. All inhabitants from the age of 40 years old and older in the Tromsø municipality were mailed an invitation. A total of 21,083 subjects (10,009 men and 11,074 women) aged 40 years old and above attended, which was 65% of those invited to participate.

### Questionnaire

As part of a more extensive questionnaire on health and illness (in total more than 300 questions), the participants completed a questionnaire with data about their use of different types of eHealth services. The following question was asked:

How often during the last year have you used the following Internet-services for information and advice on health and disease issues: Applications (‘Apps’) for smart phone or tablet?, Search engines (like Google)?, Social media (like Facebook)?, Video services (like YouTube)?

For each item, it was possible to respond either “never,” “once,” “a few times,” or “often.” Those who responded that they had used at least one of the services were subsequently asked the following question:

If you during the last year have used Internet-services for information and advice on health and disease issues, based on the information you found on the Internet: Have you decided to go to the doctor?, Have you decided not to go the doctor?, Have you discussed the information with a doctor?, Have you changed your medication without consulting a doctor?, Have you been unsure whether the treatment you have received is correct?, Have you decided to seek out complementary or alternative treatment?, Have you made lifestyle changes?, Have you felt anxiety?, Have you felt reassured?, Have you felt more knowledgeable?, Have you felt more confused?

For each of the items, it was possible to respond either “never,” “once,” “a few times,” or “often.”

The questions and their respective response options are also available online at the Tromsø Study website [51], and this was the first time these types of questions on eHealth were included in the Tromsø Study. Participants could choose to complete the questionnaire on paper or online, with most completing the questionnaire at home. However, all participants were required to attend the study center in order to participate in the study. The questionnaire (of which the eHealth questions were a small part) was supplemented with a range of tests that required people to attend in person (ie, blood tests, body measurements, electrocardiograms, ultrasounds of various organs).

### Study Sample

Variables obtained from the Tromsø 7 questionnaire included gender, age, education, occupation/work status, household income, whether the participant had seen a GP in the last year, assessment of own health, living status with a spouse, self-reported diseases, and use of the internet for finding health information. We excluded participants who had missing information on the use of the internet for health information searching (through search engines, social media, apps, or video services; n=384), and those with missing information on any of the other variables: gender, age, education, occupation, household income, GP consultation, assessment of own health, living status with spouse, and self-reported diseases (n=2202). The final analytical sample consisted of 18,497 participants (9138 men and 9359 women).

We also carried out separate analyses, including those who took health decisions (acted or not acted) following information gathering from online services (search engines, social media, apps, or video services). This subcohort included 9698 participants (4243 men and 5455 women), who had given information on these variables.

### Assessment of the Use of the Internet for Health Information and Self-Reported Diseases

Information on the use of the internet for health and participants' self-reported diseases was taken from the Tromsø 7 questionnaires. Self-reported disease conditions included: high blood pressure, heart attack, heart failure, atrial fibrillation, angina, stroke, diabetes, kidney disease, bronchitis, asthma, cancer, rheumatoid arthritis, arthrosis, migraine, psychological problems, and chronic pain. The options on these questions were “no,” or “yes,” or “yes, previously.”

The information on those (n=9698) who completed questions regarding the effect of using internet resources for health information or advice (through search engines, social media, health apps, or video services) was used in the subcohort analyses. The responses included in the present analyses were: if they had decided to visit (or not visit) the doctor, discussed information found online with a doctor, changed medication without consulting a doctor, if they had been unsure about whether the treatment they had received was correct, if they had made lifestyle changes, and if they had sought alternative or complementary treatment. The options were “never,” “once,” “a few times,” or “often.”

### Statistical Analyses

We performed multivariable logistic regression analysis with the use of the internet for health information as the dichotomous dependent variable, and gender, age, education, occupation/work status, household income, GP consultation, assessment of own health, living status with spouse, and self-reported diseases as the independent variables. The use of the internet for health information was dichotomized into never/ever by grouping those who had never used any of the resources (search engines, social media, health apps, or video services) as never, and those who had used at least one of the resources for health advice as ever. Similarly, we grouped participants who never had any of the disease conditions as never, and those participants who previously or currently had at least one condition as ever. Age was grouped into four groups: 40-49, 50-59, 60-69, and 70 years old and older. Education was grouped into four groups: primary or partly secondary education (up to 10 years of school), upper secondary education (minimum of three years), short tertiary education (college or university for less than four years), and long tertiary education (college or university for four years or more). Occupation/work status was categorized into works full time, works part-time, unemployed, housekeeper, retired, student/in military service, on disability benefit or work assessment allowance, and on family income supplement. Household income in kr per annum: less than 250,000 (<US $25,000), 250,000-450,000 (US $25,000-$45,000), 451,000-750,000 (US $45,100-$75,000), 751,000-1,000,000 (US $75,100-$100,000), and more than 1,000,000 (>US $100,000). Living status with a spouse and consultation with the GP (during the last year) were either yes or no. Assessment of own health was either very bad, bad, neither good nor bad, good, or excellent.

We checked for possible interactions between education and occupation/work status, education and income, and occupation/work status and disease condition. We further explored the relationship between the use of the internet for health information and the independent variables stratified by disease conditions (never/ever).

All *P* values were considered statistically significant at a level of <.05, and all statistical tests were two-sided. We used Stata for Windows version 15.0 (StataCorp, College Station, Texas, United States) to conduct all statistical analyses.

### Ethics

All participants gave written informed consent. The Regional Committee for Medical and Health Research Ethics approved the study (REK Nord, reference 2014/940).

## Results

### Participants’ Characteristics

Regarding age, about 60% (11,036/18,497) of the participants were within the 40-59 years old age range. Only about 15% (2759/18,497) were 70 years old or older (see Multimedia Appendix 1). The male participants had a mean age of 57.5, while the female participants had a mean age of 56.9 years. Our study sample consisted of an approximately equal proportion of men (49.4%; 9138/18,497) and women (50.6%; 9359/18,497).

For education, occupation/work status, and income, about half of the participants had tertiary education while the other half had either primary or secondary school education. The respondents were mostly in full time employment (60%; 11,188/18,497) or retired (21%; 3886/18,497). About half (51%; 9474/18,497) earned more than 750,000 kr (US $75,000) per annum, while less than 5% (890/18,497) earned 250,000 (US $25,000) or less.

A clear majority of the respondents (77.3%; 14,305/18,497) stated they were living with a spouse. As for health and psychological variables, most of the participants (80%; 14,781/18,497) had had at least one appointment with their GP during the last year, even though 70% (12,901/18,497) rated their health as excellent or good. About 73% (13,552/18,497) had had at least one of the diseases of interest in this study (see Multimedia Appendix 1).

### The Use of Electronic Health Services

One of the main findings of this study was that 52.7% (9752/18,497) of the respondents in the last year had used at least one eHealth service (ie, search engine, social media, apps, or video services) to get information and advice about health and illness (see Multimedia Appendix 1). However, the odds of using the internet for health information decreased significantly with age, with senior citizens (70 years old or older) mostly at a disadvantage (OR 0.23, 95% CI 0.20-0.28) when compared to those in the age range of 40-49 years old (see Multimedia Appendix 1).

In the multivariable analyses, we found that women had 1.58 times the odds of using internet resources (at least one of these: search engine, social media, apps, or video services) for health information when compared to men (OR 1.58, 95% CI 1.47-1.68). Also, educational level and household income positively predicted the use of the internet for health information searching. Those who had a long tertiary education had 3.77 times the odds of using internet resources to look for health information compared to those who only had primary or partly secondary school education (OR 3.77, 95% CI 3.40-4.19). Similarly, those who earned the most were significantly at increased odds of using internet resources (OR 2.19, 95% CI 1.77-2.70) when compared to those who earned the least. Occupation or work status did not predict the use of internet resources for health information. However, those on disability benefits and other family welfare benefits had 1.71 times the odds of using the internet for health information when compared to those who worked full time (OR 1.71, 95% CI 1.05-2.78).

In regard to living with a spouse, those participants had 0.88 times the odds of using the internet for health information when compared to those who were not living with a spouse (OR 0.88, 95%CI 0.80-0.97). We also found that those who had consulted their GP in the last year had 1.66 times the odds of using internet resources for health information compared to those who had not consulted their GP. Similarly, those who had ever had at least one of the diseases of interest were at increased odds of using the internet for health information (OR 1.29, 95% CI 1.18-1.41). Intriguingly, assessment of own health did not predict the use of internet resources for health information searching (see Multimedia Appendix 1).

### Taking Action After Obtaining Information

About 58% (5624/9696) of those who answered this question took some form of action after having obtained information about health and illness on the internet (see Multimedia Appendix 1). The action taken varied from deciding to see a doctor or not to see a doctor, discussing the information with a doctor, changing a medication without consulting a doctor, questioning previous treatment, deciding to seek alternative or complementary treatment, or changing lifestyle.

In the multivariable analyses of the subcohort (n=9698) who made health decisions following use of the internet for health information searching, we found that similar to the use of internet resources for health information, the odds of making health-related decisions following use decreased with age. Those aged 70 years old and above had nearly half the odds of making health-related decisions/actions when compared to those that were 40-49 years old (OR 0.58, 95% CI 0.45-0.75). Also, we found that women had 1.16 times the odds of making health-related decisions following the use of internet resources when compared to men (OR 1.16, 95% CI 1.07-1.27).

Regarding education and income, educational level positively predicted making health-related decisions following the use of internet resources for health information. Those with a long tertiary education had 1.65 times the odds of making health-related decisions following use when compared to those who had primary or partly secondary school education (OR 1.65, 95% CI 1.42-1.92). However, household income did not significantly predict health-related decision-making following the use of internet resources.

Unlike in the use of the internet for health information, not living with a spouse did not significantly predict health-related decision-making following the use of internet resources. Additionally, those who had consulted their GP in the last year (OR 1.58, 95% CI 1.41-1.77) and those who had had at least one of the diseases of interest (OR 1.26, 95% CI 1.41-1.39) had increased odds of taking health actions following internet resources use (see Multimedia Appendix 1). We found that occupation or work status and assessment of own health did not predict health-related decision-making following the use of internet resources, which is similar to our findings concerning the use of internet resources for health information.

## Discussion

### Use of Electronic Health and Predictors of Use

#### Overview

We found that approximately half of the respondents had used some form of eHealth during the last year. This figure is lower than what has been suggested in some prior studies [8,12,13]. However, as our study consisted of a sample of the population that is 40 years old and older, we did not include the younger generation that is likely to have a higher internet use. Moreover, our sample was from the general population and not restricted to internet users, which in part may explain the lower rate of eHealth use in our study [34].

#### Age

Younger age was a significant positive predictor of eHealth use, in line with the findings of several prior studies [8,13,17,35]. Unsurprisingly, the younger are more knowledgeable and comfortable with eHealth services, as more young people use online services [13]. This finding is also consistent with prior studies that have determined that younger users are more accurate and have more attention to detail when using eHealth resources [52,53]. Moreover, older adults may have barriers to technology use because of perceived complexity that may limit confidence and interest in engaging with the technology [54]. Internet use has been shown to be especially lower among those above 75 years of age [55]. Increased age has been related to lower rates of shared decision-making in traditional health services [56]. As the use of eHealth could be related to shared decision-making (ie, informed and empowered patients are more likely to be interested in shared decision-making), lower eHealth use in the oldest age groups might in part be explained by a lower rate of shared decision-making among the most senior.

Some prior studies have suggested higher rates of use in the older age groups than we did in the present study [26,39,57], but differences in the range of the age groups between studies make direct comparisons challenging. Another possible cause of differences in results regarding use could be related to whether participants are sampled from the general population or from groups of internet users (eHealth use is likely to be higher among internet users). It might be of concern that those who are in the age groups most in need of health services (ie, the elderly, who typically are iller than the younger generation) use these services less. Helping elderly patients find appropriate online information and better adapting the information to suit their needs (in terms of content, style, readability), might increase use [58]. However, this age-related difference might diminish as more older people are accessing the internet. In the EU in 2012, 42% of those in the 55-74 years old age group were regular users [4], and in 2015 a total of 58% of US senior citizens were online [3]. In summary, our finding that the younger use eHealth more often is supported by prior literature.

#### Gender

We also found that being a woman was a significant predictor of use, in line with previous findings [8,9,13,35,59]. This gender difference in eHealth use could be explained by the fact that women are more engaged in health care in general because they often serve as family caregivers, holders of health information, and care managers [60-62], and also have a higher use of social media [63], possibly because of gender roles. Thus, prior studies have suggested that women are more active eHealth users than men, and our findings strengthen this idea.

#### Education, Occupation/Work Status, and Income

We found that having a higher education positively predicted the use of eHealth. Higher education has also previously been shown to predict eHealth use [9,13,17,35], a finding that could be related to higher health literacy (ie, “the degree to which individuals have the capacity to obtain, process, and understand basic health information and services needed to make appropriate health decisions” [64]) and higher patient engagement (ie, to “promote and support active patient and public involvement in health and healthcare and to strengthen their influence on healthcare decisions, at both the individual and collective level” [65]) in health among the higher educated.

A review study has found that patients’ engagement with digital health decreases with higher age and lower health literacy [66]. People working in some professions, such as those who routinely use the internet at work [67], may have more time to search for health information. Some prior studies have suggested that occupation and work status might be of importance [17,68], although the finding has not been consistent [8]. In our study, occupation and work status were, overall, not significant predictors. As our study has a large sample and consequently high statistical power, our finding may suggest that occupation and work status are less significant central predictors, at least in a Nordic setting. In line with a prior study [13], we found that increased household income also positively predicted eHealth use. In summary, while higher education and income positively predicted eHealth use in our study, we did not find a similar relationship for occupation and work status.

#### Living With a Spouse

While loneliness is known to increase the risk of death [69], living with a spouse reduces rates of illness and death from a range of illnesses [70]. These positive health effects have been associated with the support spouses offer each other [70]. An American study found that 39% of adults were caregivers, and many of these cared for their partner [71]. Those who search online for health information for others are more likely to live in households with others [72]. Drawing on some prior research [12,71], one could assume that spouses might have a higher use for eHealth because they might be searching for information relating to their partner. However, living with a spouse negatively predicted eHealth use in our study, possibly because spouses might either inform or comfort each other in such a way that the need for health information from other sources is reduced. Our finding that living with a spouse negatively predicted eHealth use thus stands somewhat in contrast to what some prior researchers have found.

#### Health and Psychological Variables

Health and psychological variables have, to varying degrees, been found to predict health-related internet use [17]. In our study, having seen a GP in the last year positively predicted eHealth use. Respondents who stated that they had (or previously had) an illness also used eHealth more, while self-reported health status was not a significant predictor. Poor or fair self-reported health or chronic illness has predicted health-related internet use in some other studies [8,31,35,73]. Fox and Duggan [12] found that living with a chronic disease had an independent negative effect on internet access in the United States. However, internet users with chronic illness were more likely to gather online information about medical problems. As a high percentage of Norwegians have internet access, one might consequently expect that having an illness would predict eHealth use, as we found in the present study. Thus, having an illness or having seen a GP positively predicted eHealth use in our study, as might be expected in a country with a very high internet access rate.

### Taking Action Based on Online Information

#### Overview

The finding that about 6/10 acted based on information gained from using eHealth services suggests that health information on the internet plays a surprisingly important role in people’s decision-making processes regarding their health care. The action taken included deciding to see a doctor or not to see a doctor. It is not surprising that health information may help people make such a decision, and many people probably search for health information online to get a basis for deciding whether they need professional help or not.

Other actions taken were discussing the information with a doctor, changing medication without talking with a doctor, deciding to see an alternative practitioner, or changing lifestyle. Prior studies have suggested that many patients obtain information from the internet that they want to discuss with their doctor [8,39,74], and sometimes such information may lead the patient to question the diagnosis or the treatment given by the doctor [74]. Lifestyle advice, such as advice relating to starting to exercise, stopping smoking, or dieting, is one of the most popular types of health-related information that people seek on the internet [8]. Many people use complementary and alternative medicine (CAM) and search for information related to CAM online [75,76]. In summary, we found that online health information was important for many in making decisions relating to their health.

#### Gender, Age, Education, and Health

Many of the same variables were of importance to acting on the information as to accessing it in the first place, and being a woman, being of younger age, having higher education, having seen a GP in the last year, and having ever had an illness all predicted taking some form of action. Searching for information and acting on this information are qualitatively different processes. However, both behaviors are determined by many of the same variables. Household income was not a predictor of acting on the information, possibly because health care is covered by national insurance in Norway. Thus, searching for information and acting on this information were predicted by mostly the same variables.

### Electronic Health and Traditional Health Services

eHealth was associated with the use of traditional health services (ie, having seen the GP during the last year). It is possible that using online health information may increase traditional health service consumption. We know that health-related information on the internet, on social media, and video services may be wrong, misleading, or biased [17,77-79] and that this information may generate increased uncertainty or anxiety among users and result in a need for clarification and interpretation [27,29,39,80,81]. Doctors are still considered the most reliable source of information [82], and most (88%) Norwegians still favor seeing their GP face-to-face [8]. In paper 4 [49] in this series, we further explore the association between eHealth use and an increase in GP visits in Norway.

### Reducing the Digital Divide

We have found that higher age, being male, and having lower education, not having an illness, and not having seen a GP in the last year were associated with a lower use of eHealth services. We do not know why some subgroups used the internet less for health purposes. We suggest that a lower degree of engagement in health, in general, might explain some of the differences in eHealth use. Furthermore, some may not access eHealth services because they are unaware of the service [83], because they find it difficult to use [84], because they find it irrelevant [85], or because they find it difficult to trust. Many websites with quality health information have low readability and may be difficult to understand for people with low literacy levels [86]. The fact that some groups use online health information and eHealth tools less often suggests that these services and tools need to be matched to the eHealth-use abilities of these underserved groups [84]. Suggesting appropriate sources of online information and using other types of health information, including traditional offline media, might be considered as a strategy for reaching those who use the internet less for health purposes [13].

### Strengths and Limitations

This is the first representative, large-scale, population-based study of eHealth use in Norway. We have given a representative picture of the use of eHealth in a population 40 years old and older, predictors of eHealth use, and predictors of taking action following the use of eHealth services. There are important differences in the organization and funding of health care in the United States, Norway, and much of Europe. Despite these differences, lower age, female gender, higher educational level, and having a chronic illness seemed to predict increased eHealth use both in the United States and in Norway.

There are some central limitations to this study. There might be a self-selection bias because not everyone who was invited chose to participate. As this study was based on cross-sectional data obtained from questionnaires, there is a possibility of recall bias (ie, that participants either underestimated or overestimated their use of eHealth or their actions taken). However, the validity and reproducibility of self-reported (ie, recalled) findings from the Tromsø Study have been reported as quite high and of sufficient quality for epidemiological research [87,88]. Also, due to the cross-sectional design of the study, we are unable to establish causation. There is also a risk that there might be unmeasured confounding variables. We did not have variables on patients’ trust in online information or on patients’ literacy levels. One variable used was self-reported health, which has been shown to be influenced by socioeconomic class [89]. The questionnaire did not include more detailed questions about how people use and experience different eHealth services, and this issue is an important avenue for future large-scale studies.

### Conclusions

About half of respondents used some form of eHealth in the last year, and about 6/10 of this half used the information to take some form of action. The use of eHealth was associated with the use of traditional health services. This study has provided new knowledge about the importance of the internet, social media, apps, and online videos for health information and how this information impacts patients and the general public. While one might hope that eHealth services can benefit those most in need, the present study suggests that it is those with the most resources, the highly educated and well-off, that consume eHealth services the most. Being in poor health did not predict the use of online health information. Clinicians should be aware that many patients above 40 years of age use eHealth to find information about health and illness, and that they also often act on this information [26,39,57]. The provision of high-quality eHealth services should, therefore, be a priority for clinicians and health service providers. Clinicians should be aware that the use of eHealth sometimes has important medical consequences, such as when patients decide not to visit their doctor or to stop taking their medication without consulting their doctor. Some groups of patients, such as the oldest and those with little education, appear to use eHealth less than other groups, possibly because the services are not adequately matched to their needs. Clinicians might consider recommending adapted online or paper-based information specifically for these groups.

## Appendix

Multimedia Appendix 1 Study tables.

## Abbreviations

CAM: complementary and alternative medicine
eHealth: electronic health
EU: European Union
GP: general practitioner
HINTS: Health Information National Trends Survey
Tromsø 7: seventh survey of the Tromsø Study
