# Supplementary material for: Electronic Health Use in a Representative Sample of 18,497 Respondents in Norway (The Seventh Tromsø Study - Part 1): Population-Based Questionnaire Study
Source: JMIR Med Inform. 2020 Mar 5;8(3):e13106. doi: 10.2196/13106 (PMC7082740; doi:10.2196/13106)
Supplement: Multimedia Appendix 1 [file medinform_v8i3e13106_app1.docx]

Appendix: Tables 1-3

**Table1: Characteristics of the Tromsø 7 study sample (N=18,497)**

| Characteristics |  | Counts, n (%) |
| --- | --- | --- |
|  |  |  |
| **Gender** |  |  |
|  | Women | 9,359 (50.6) |
|  | Men | 9,138 (49.4) |
| **Age groups** |  |  |
|  | 40-49 | 5,499 (29.7) |
|  | 50-59 | 5,537 (29.9) |
|  | 60-69 | 4,702 (25.4) |
|  | 70 & above | 2,759 (14.9) |
| **Education** |  |  |
|  | Primary/partly secondary | 3,942 (21.3) |
|  | Upper secondary | 5,149 (27.8) |
|  | Tertiary, short | 3,690 (20.0) |
|  | Tertiary, long | 5,716 (30.9) |
| **Occupation/work status** |  |  |
|  | Works full time | 11,188 (60.5) |
|  | Works part time | 1,504 (8.1) |
|  | Unemployed | 121 (0.7) |
|  | Housekeeping | 106 (0.6) |
|  | Retired | 3,886 (21.0) |
|  | Student/military service | 52 (0.3) |
|  | Disability benefit and other family welfare benefits | 1,640 (8.9) |
| **Household income ‘000** |  |  |
|  | < or = 250 NOK/25 USD | 890 (4.8) |
|  | 250-450 NOK/25-45 USD | 2,787 (15.1) |
|  | 451-750 NOK/45,1-75 USD | 5,346 (28.9) |
|  | 751-1000 NOK/75,1-100 USD | 4,576 (24.7) |
|  | > 1000 NOK/100 USD | 4,898 (26.5) |
| **GP consultation** |  |  |
|  | No | 3,716 (20.1) |
|  | Yes | 14,781 (79.9) |
| **Assessment of own health** |  |  |
|  | Very bad | 55 (0.3) |
|  | Bad | 910 (4.9) |
|  | Neither good nor bad | 4,631 (25.0) |
|  | Good | 10,118 (54.7) |
|  | Excellent | 2,783 (15.1) |
| **Live with spouse** |  |  |
|  | No | 4,192 (22.7) |
|  | Yes | 14,305 (77.3) |
| **Self-reported diseases** |  |  |
|  | Never had any | 4,945(26.7) |
|  | Ever | 13,552 (73.3) |
| **Use of internet for health (at least one of these: search engine, social media, apps, or video services)** |  |  |
|  | Never | 8,745 (47.3) |
|  | Ever | 9,752 (52.7) |

**Table 2: Factors affecting the use of the Internet for health information: The Tromsø Study (N= 18,497)**

| Potential predictors of internet use for health information |  | Use of internet for health (at least one of these: *search engine, social media, apps, or video services)* | | Multivariable logistic regression |
| --- | --- | --- | --- | --- |
|  |  | Ever use | Never | Odds ratios (95% CI)^a^ |
|  |  |  |  |  |
| **Gender** |  |  |  |  |
|  | Men | 4,262 | 4,876 | 1.00 |
|  | Women | 5,490 | 3,869 | 1.58 (1.47-1.68)^b^ |
| **Age groups** |  |  |  |  |
|  | 40-49 | 3,835 | 1,664 | 1.00 |
|  | 50-59 | 3,190 | 2,347 | 0.63 (0.58-0.69)^b^ |
|  | 60-69 | 2,018 | 2,684 | 0.38 (0.35-0.42)^b^ |
|  | 70 & above | 709 | 2,050 | 0.23 (0.20-0.28)^b^ |
| **Education** |  |  |  |  |
|  | Primary/partly secondary | 1,142 | 2,800 | 1.00 |
|  | Upper secondary | 2,484 | 2,665 | 1.79 (1.63-1.97)^b^ |
|  | Tertiary, short | 2,196 | 1,494 | 2.76 (2.48-3.06)^b^ |
|  | Tertiary, long | 3,930 | 1,786 | 3.77 (3.40-4.19)^b^ |
| **Occupation/work status** |  |  |  |  |
|  | Works full time | 6,729 | 4,459 | 1.00 |
|  | Works part time | 867 | 637 | 0.87 (0.67-1.14) |
|  | Unemployed | 76 | 45 | 1.20 (0.52-2.78) |
|  | Housekeeping | 36 | 70 | 2.53 (0.90-7.09) |
|  | Retired | 1,156 | 2,730 | 1.11 (0.90-1.39) |
|  | Student/military service | 37 | 15 | 1.54 (0.51-4.63) |
|  | Disability benefit and other family welfare benefits | 851 | 789 | 1.71 (1.05-2.78)^c^ |
| **Household income ‘000** |  |  |  |  |
|  | < or = 250 NOK/25 USD | 233 | 657 | 1.00 |
|  | 250-450 NOK/25-45 USD | 1,127 | 1,660 | 1.67 (1.39-2.01)^b^ |
|  | 451-750 NOK/45,1-75 USD | 2,596 | 2,750 | 1.84 (1.52-2.22)^b^ |
|  | 751-1000 NOK/75,1-100 USD | 2,671 | 1,905 | 2.16 (1.76-2.64)^b^ |
|  | > 1000 NOK/100 USD | 3,125 | 1,773 | 2.19 (1.77-2.70)^b^ |
| **GP consultation** |  |  |  |  |
|  | No | 1,733 | 1,983 | 1.00 |
|  | Yes | 8,019 | 6,762 | 1.66 (1.53-1.80)^b^ |
| **Assessment of own health** |  |  |  |  |
|  | Very bad | 29 | 26 | 1.00 |
|  | Bad | 529 | 381 | 1.23 (0.67-2.27) |
|  | Neither good nor bad | 2,256 | 2,375 | 0.91 (0.50-1.66) |
|  | Good | 5,391 | 4,727 | 0.89 (0.49-1.62) |
|  | Excellent | 1,547 | 1,236 | 0.79 (0.43-1.45) |
| **Live with spouse** |  |  |  |  |
|  | No | 2,115 | 2,077 | 1.00 |
|  | Yes | 7,637 | 6,668 | 0.88 (0.80-0.97)^c^ |
| **Self-reported diseases** |  |  |  |  |
|  | Never had any | 2,542 | 2,403 | 1.00 |
|  | Ever | 7,210 | 6,342 | 1.29 (1.18-1.41)^b^ |

^a^ 95% CI = 95% confidence interval, ^b^ significant at *P* < 0.001, ^c^ significant at *P* < 0.05

**Table 3: Factors affecting whether Internet info led to action (N= 9,698)**

| Potential predictors of action following use of internet for health information |  | Internet info led to action (info from at least one of these: *search engine, social media, apps, or video services*) | | Multivariable logistic regression |
| --- | --- | --- | --- | --- |
|  |  | Ever use | Never | Odds ratios (95% CI)^a^ |
|  |  |  |  |  |
| **Gender** |  |  |  |  |
|  | Men | 2,323 | 1,920 | 1.00 |
|  | Women | 3,301 | 2,154 | 1.16 (1.07-1.27)^d^ |
| **Age groups** |  |  |  |  |
|  | 40-49 | 2,352 | 1,468 | 1.00 |
|  | 50-59 | 1,841 | 1,341 | 0.84 (0.76-0.92)^b^ |
|  | 60-69 | 1,068 | 933 | 0.64 (0.57-0.73)^b^ |
|  | 70 & above | 363 | 332 | 0.58 (0.45-0.75)^b^ |
| **Education** |  |  |  |  |
|  | Primary/partly secondary | 573 | 556 | 1.00 |
|  | Upper secondary | 1,411 | 1,063 | 1.35 (1.17-1.57)^b^ |
|  | Tertiary, short | 1,335 | 852 | 1.72 (1.47-2.01)^b^ |
|  | Tertiary, long | 2,305 | 1,603 | 1.65 (1.42-1.92)^b^ |
| **Occupation/work status** |  |  |  |  |
|  | Works full time | 3,838 | 2,871 | 1.00 |
|  | Works part time | 520 | 343 | 1.03 (0.88-1.21) |
|  | Unemployed | 46 | 28 | 1.13 (0.70-1.84) |
|  | Housekeeping | 16 | 19 | 0.69 (0.35-1.38) |
|  | Retired | 606 | 532 | 1.10 (0.90-1.36) |
|  | Student/military service | 25 | 12 | 1.28 (0.64-2.58) |
|  | Disability benefit and other family welfare benefits | 573 | 269 | 1.33 (1.11-1.59)^d^ |
| **Household income ‘000** |  |  |  |  |
|  | < or = 250 NOK/25 USD | 143 | 80 | 1.00 |
|  | 250-450 NOK/25-45 USD | 667 | 453 | 0.88 (0.65-1.20) |
|  | 451-750 NOK/45,1-75 USD | 1,520 | 1,060 | 0.85 (0.62-1.15) |
|  | 751-1000 NOK/75- 100 USD | 1,539 | 1,116 | 0.80 (0.57-1.10) |
|  | > 1000 NOK/100 USD | 1,755 | 1,365 | 0.77 (0.55-1.07) |
| **GP consultation** |  |  |  |  |
|  | No | 805 | 923 | 1.00 |
|  | Yes | 4,819 | 3,151 | 1.58 (1.41-1.77)^b^ |
| **Assessment of own health** |  |  |  |  |
|  | Very bad | 17 | 12 | 1.00 |
|  | Bad | 380 | 145 | 2.05 (0.95-4.47) |
|  | Neither good nor bad | 1,399 | 832 | 1.53 (0.72-3.27) |
|  | Good | 3,051 | 2,319 | 1.26 (0.59-2.68) |
|  | Excellent | 777 | 766 | 1.04 (0.48-2.24) |
| **Live with spouse** |  |  |  |  |
|  | No | 1,269 | 7834 | 1.00 |
|  | Yes | 4,355 | 3,240 | 0.99 (0.88-1.13) |
| **Self-reported diseases** |  |  |  |  |
|  | Never had any | 1,287 | 1,243 | 1.00 |
|  | Ever | 4,337 | 2,831 | 1.26 (1.14-1.39)^b^ |

^a^ 95% CI = 95% confidence interval, ^b^ significant at *P* < 0.001, ^d^ significant at *P* < 0.005
